# Cell-Autonomous Requirement for *Rx* Function in the Mammalian Retina and Posterior Pituitary

**DOI:** 10.1371/journal.pone.0004513

**Published:** 2009-02-20

**Authors:** Olga Medina-Martinez, Felipe Amaya-Manzanares, Chaomei Liu, Marisela Mendoza, Rina Shah, Li Zhang, Richard R. Behringer, Kathleen A. Mahon, Milan Jamrich

**Affiliations:** 1 Department of Molecular and Human Genetics, Baylor College of Medicine, Houston, Texas, United States of America; 2 Department of Molecular and Cellular Biology, Baylor College of Medicine, Houston, Texas, United States of America; 3 Department of Molecular Genetics, University of Texas, M. D. Anderson Cancer Center, Houston, Texas, United States of America; Katholieke Universiteit Leuven, Belgium

## Abstract

*Rx* is a paired-like homeobox gene that is required for vertebrate eye formation. Mice lacking *Rx* function do not develop eyes or the posterior pituitary. To determine whether *Rx* is required cell autonomously in these tissues, we generated embryonic chimeras consisting of wild type and *Rx−/−* cells. We found that in the eye, *Rx*-deficient cells cannot participate in the formation of the neuroretina, retina pigment epithelium and the distal part of the optic stalk. In addition, in the ventral forebrain, *Rx* function is required cell autonomously for the formation of the posterior pituitary. Interestingly, *Rx−/−* and wild type cells segregate before the morphogenesis of these two tissues begins. Our observations suggest that *Rx* function is not only required for the morphogenesis of the retina and posterior pituitary, but also prior to morphogenesis, for the sorting out of cells to form distinct fields of retinal/pituitary cells.

## Introduction

Several homeobox genes play important roles in the patterning of the vertebrate forebrain. One of these is the paired-like homeobox gene *Rx* [1]–[3]. *Rx* plays a critical role in vertebrate eye development, as mutations in this gene cause the loss of or abnormal formation of the eye in several species [2], [4]–[11]. *Rx* transcription begins in the anterior neural plate of gastrula embryos [1]–[3], in an area that gives rise to the retina, optic stalk and ventral hypothalamus.

Retinal formation begins with the evagination of retinal progenitor cells. These cells initially form the optic vesicle and later the optic cup, which consists of the neuroretina and the retina pigment epithelium (RPE). The retina remains connected to the brain through the optic stalk. The evaginating neuroectoderm induces lens formation in the overlying surface ectoderm. Mice lacking *Rx* function do not develop optic vesicles or optic cups and consequently do not develop a neuroretina, a RPE or an optic stalk [2]. Since ocular neuroectoderm is necessary for lens formation, *Rx*-deficient embryos do not form a lens, despite the fact that *Rx* is not expressed in the surface ectoderm [2], [10]–[12]. In medaka, an intronic insertion in *Rx3* is the cause of the temperature sensitive *eyeless* (*el*) mutant [13]. This mutation results in the transcriptional repression of *Rx3* and is responsible for the lack of evagination of the optic vesicle [6], [13]. In zebrafish, the eyeless phenotype in the *chokh* mutant is caused by a mutation in the homeobox region of *Rx3* [5]. Finally, in human, mutations in *Rx/RAX* lead to anophthalmia [8].

In addition to eye development, *Rx* function is required for the normal development of the ventral forebrain [14]. During normal development, the ventral forebrain gives rise to the hypothalamo-pituitary axis. The posterior pituitary forms from the ventral hypothalamus through evagination. The ventral hypothalamus and posterior pituitary are instrumental in the induction and correct differentiation of the anterior pituitary that is formed from the head surface ectoderm [15]–[19].

We have observed previously that the ventral neuroectoderm is very thin [14] and does not undergo proper morphogenesis in *Rx*-deficient mouse embryos. The posterior pituitary cannot be detected in these animals at the time of birth and Rathke's pouch, the precursor of the anterior pituitary, begins to form, but does not differentiate properly [14]. The ventral hypothalamus is a rich source of signaling molecules and it has been shown that signaling from this region is necessary for the formation of the anterior pituitary. *Bmp4* and *Fgfs* are expressed in the ventral hypothalamus and are required for induction of the pouch primordium and formation of a definitive Rathke's pouch, respectively [20]–[23]. Members of the Sonic hedgehog (SHH), Wnt, and Notch signaling pathways have also been implicated in these processes. Therefore, it is likely that abnormal differentiation of the anterior pituitary in *Rx* mutant mice is the consequence of abnormal development/signaling from the hypothalamus. Interestingly, the developing retina (optic cup) and the ventral diencephalon that gives rise to the hypothalamus/posterior pituitary, display similarities in morphogenesis and gene expression. The retina, as well as the posterior pituitary, evaginates from the neuroectoderm and both initially express *Rx*. The optic cup is instrumental in the induction, invagination and differentiation of the lens from the head surface ectoderm. Similarly, the ventral hypothalamus is instrumental in the induction, invagination and the differentiation of Rathke's pouch (the anterior/intermediate pituitary), which is also derived from the surface ectoderm. It is therefore possible that the formation of both tissue pairs (retina-lens and hypothalamus-anterior pituitary) is regulated by similar developmental and molecular mechanisms. Indeed, besides *Rx*, there are several homeodomain transcription factors (i.e. *Pax6*, *Six3*, *Optx2*, *Lhx2*, *Foxl2*), as well as signaling molecules (SHH, BMP4, FGF-8, -10, Wnt and Notch pathway members) expressed during eye development, that are also expressed in analogous ways in the development of the hypothalamic-pituitary axis. Furthermore, the intriguing observation that the adenohypophysis is transformed into an ectopic lens in the zebrafish midline mutants *you-too (yot)* and *iguana (igu)* by a mutation in *Gli2* gene, is additional evidence for developmental and molecular commonality [24].

While it is known that *Rx* expression is required for the formation of the retina and the hypothalamo-pituitary axis in mouse embryos, it is not known whether *Rx* function is required cell autonomously in these tissues. A cell autonomous requirement for *Rx3* function for the morphogenesis of the retina has been reported in medaka and zebrafish [25]–[27], but the mechanism of retinal formation is significantly different in mammals and teleosts [25]. The question of cell autonomous function of *Rx* in the retina and the hypothalamo-pituitary axis has yet to be examined in mammals.

To determine whether *Rx* expression is required cell autonomously for the formation of the retina and the hypothalamo-pituitary axis in mouse embryos, we analyzed the behavior of *Rx*-deficient cells in embryonic chimeras consisting of wild type and *Rx−/−* cells. We also characterized the changes in gene expression during pituitary development in *Rx*-deficient embryos. Results of our studies show that *Rx* function is required cell-autonomously for the formation of the retina and the posterior pituitary and that *Optx2* is the common target of *Rx* during the development of both tissues.

## Results

**Figure 1:**
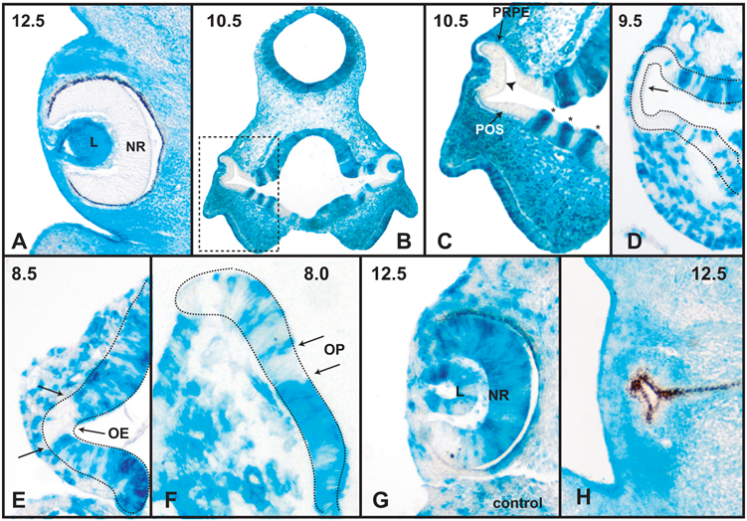
Retinal formation in chimeric embryos. (A) Section through an E12.5 chimeric embryo demonstrating that the neuroretina does not contain any *Rx*-deficient (blue) cells. (B) Section through an E10.5 chimeric embryo. Boxed region is enlarged in C. (C) Section through an E10.5 chimeric embryo visualizing the absence of *Rx*-deficient cells in the presumptive neuroretina (arrowhead), RPE and distal optic stalk. Columns of *Rx*-deficient cells in the proximal optic stalk are labeled with an asterisk. (D) Section through an E9.5 chimeric embryo showing the absence of *Rx*-deficient cells in the evaginating optic vesicle (arrow). (E) Section through an E8.5 chimeric embryo showing predominantly wild type cells in the evaginating optic neuroectoderm. Two arrows indicate the sharp boundaries between the wild type cells (white) and *Rx−/−* cells (blue). (F) Section through an E8.0 chimeric embryo showing the distribution of wild type and *Rx*-deficient cells in the optic pit. (G) A control chimera. Section through an E12.5 chimeric embryo generated from wild type (white) and *Gt(ROSA)26Sor/J* (blue) cells demonstrating that *Gt(ROSA)26Sor/J* cells that are wild type for *Rx*, participate in retinal formation. (H) Section through an E12.5 chimeric embryo generated from wild type (white) and *Rx−/−* cells demonstrating that in the presence of only few wild type cells, the entire optic cup develops into an RPE. No lens is present in this case. L – lens, NR – neuroretina, OE – optic evagination, OP – optic pit, POS - presumptive optic stalk, PRPE – presumptive retina pigment epithelium.

Our previous experiments have shown that *Rx* is required for eye formation in mouse. In the absence of *Rx*, the optic structures such as the optic sulci, optic vesicle and optic cup do not form and the expression of retina and lens specific markers cannot be detected [2], [14]. To determine whether *Rx* function is required cell autonomously during retinal development, we generated embryonic chimeras consisting of wild type and *Rx−/−^lacZ/+^* cells. Chimeric embryos bearing both wild-type (white) and varying amounts of mutant cells (blue) were examined to determine whether *Rx* mutant cells could contribute to eye structures in a wild type environment. We analyzed chimeras with a very high contributions of *Rx−/−^lacZ/+^* cells, but we did not observe any *Rx−/−^lacZ/+^* cells in retinal structures. As figure 1A shows, at E12.5 the neuroretina is totally devoid of *Rx−/−* cells, while most of the cells surrounding the eye, as well as the lens cells, are *Rx*−/−. Since the pigmentation of the retina pigment epithelium (RPE) makes it difficult to determine whether the cells of the RPE are wild type or *Rx−/−*, we analyzed chimeric embryos at E10.5 – before the pigmentation of RPE appears (Fig. 1B, C). Sections through an E10.5 chimeric embryo show that *Rx−/−* cells do not participate in the formation of the presumptive neuroretina, RPE or the distal part of the optic stalk (Fig. 1C). These observations demonstrate that there is a cell-autonomous requirement for *Rx* function in these retinal structures. Interestingly, in the transition zone from the neural tube to the optic stalk, the wild type cells and *Rx*-deficient cells can coexist, but they sort out into columns of cells based on their genetic makeup. The columns consisting of different cell types display different morphology, with *Rx*-deficient columns cells always being wider than the columns of wild type cells. The likely explanation for the different thickness of tissue of different genetic makeup is that the wild type cells can participate in the convergent extension of the optic stalk, while the *Rx−/−* cells cannot. The lack of *Rx−/−* cells in the evaginating optic vesicle can be observed at E9.5, when the vesicle is devoid of *Rx*-deficient cells (Fig. 1D). A relatively sharp boundary between the evaginating cells of the presumptive optic vesicle and the surrounding cells can be detected as early as E8.5 (Fig. 1E), the beginning of evagination of the ocular neuroectoderm. Importantly, at E8.0, the optic pit contains mostly wild type cells (Fig. 1F), suggesting that *Rx−/−* cells are depleted from this region even before the morphogenesis of the retinal structures begins. It should be pointed out that because of the lack of molecular markers specifically expressed in this region, the optic pit was identified based on its morphology. In contrast, in control chimeras generated from wild type (white) and *Gt(ROSA)26Sor/J* cells that were *Rx+/+^lacZ/+^*, both cell types contributed equally to the formation of the neuroretina (Fig. 1G).

Interestingly, when the contribution of wild type cells in the chimeric embryo was very low, the wild type cells developed into an optic vesicle consisting only of RPE (Fig. 1H). Lenses were not observed in these embryos, suggesting that this optic vesicle cannot induce lens formation.

**Figure 2:**
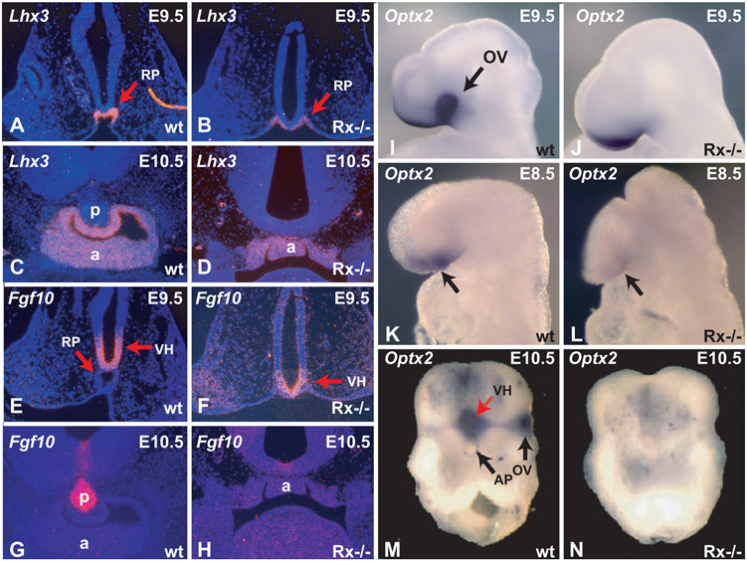
Expression of pituitary specific genes in wild type and *Rx*-deficient embryos. (A) *Lhx3* expression in Rathke's pouch of an E9.5 wild type embryo. (B) *Lhx3* expression in Rathke's pouch of an E9.5 *Rx*-deficient embryo. (C) *Lhx3* expression in the anterior pituitary of an E10.5 wild type embryo. (D) *Lhx3* expression in the dysmorphic anterior pituitary of an E10.5 *Rx*-deficient embryo. (E) Expression of *Fgf10* in the ventral hypothalamus of an E9.5 wild type embryo. (F) Expression of *Fgf10* in the ventral hypothalamus of an E9.5 *Rx*-deficient embryo. (G) Expression of *Fgf10* in the posterior pituitary of an E10.5 wild type embryo. (H) *Fgf10* expression in an *Rx*-deficient embryo. (I) *Optx2* expression in the head of an E9.5 wild type embryo. (J) *Optx2* expression in the head of an E9.5 *Rx−/−* embryo. (K) *Optx2* expression in the neuroectoderm and head surface ectoderm of an E8.5 wild type embryo. (L) *Optx2* expression in the head surface ectoderm of an E8.5 *Rx−/−* embryo. (M) *Optx2* expression in the ventral hypothalamus and optic vesicle of an E10.5 wild type embryo. (N) Lack of *Optx2* expression in an E10.5 *Rx−/−* embryo. a – anterior pituitary, p – posterior pituitary, OV – optic vesicle, RP- Rathke's pouch, VH – ventral hypothalamus.

Since our previous results indicated that the formation of the hypothalamo-pituitary axis was also strongly affected in *Rx−/−* embryos [14], we investigated the role of *Rx* in the formation of the pituitary. During normal development, the primordium of the anterior and intermediate lobes of the pituitary gland (adenohypophysis) is first visible as a thickened region of the midline oral ectoderm that lies adjacent to the floor of the diencephalon or future hypothalamus. In the mouse embryo, this ectoderm invaginates at approximately 8.5–9 days to form Rathke's pouch (RP) (Fig. 2A), which detaches from the oral ectoderm by 12–12.5 days [for review see reference 18]. At the same time, the infundibulum, an outpocketing of the diencephalon, evaginates to give rise to the neurohypophysis or the posterior lobe of the pituitary (Fig. 2C). The neurohypophysis contains the axon terminals of the neurosecretory neurons that reside in the hypothalamus. These two tissues lie in direct contact during this time, and a large body of evidence indicates that signals from the neuroectoderm of the ventral diencephalon (hypothalamus) are required for the induction, specification and commitment of Rathke's pouch and pituitary cell lineages [for review see 19]. In *Rx−/−* embryos, there is no evidence for evagination of the ventral neuroectoderm to form the posterior pituitary (Fig. 2D). Rathke's pouch initially forms in these mutants [14], but it becomes severely dysmorphic (Fig. 2D) and cannot be visually identified at birth (not shown). Despite the presence of clefts, the pouch tissue remains as a multilayered sheet; it does not ever detach from the oral ectoderm and remains in contact with the oral cavity (Fig. 2D). Ultimately, the pituitary and ventral hypothalamic tissue are extruded into the oral cavity about the same time that the palate would normally be closing (not shown).

In order to better define the molecular steps that lead from *Rx* expression to the proper development of the hypothalamus and formation of the posterior pituitary and to better understand the development of the pituitary gland *in Rx−/−* embryos, we examined the expression of several genes during the development of the anterior and posterior pituitary in wild type and *Rx*-deficient embryos. Here we present the comparison of *Lhx3*, *Fgf10* and *Optx2* expression in wild type and *Rx−/−* embryos, as the changes in the expression of these three genes best demonstrate the morphological and molecular consequences of the absence of *Rx* expression. *Lhx3* is a specific marker of anterior pituitary development. It is a LIM-homeobox gene, expressed as soon as Rathke's pouch forms and its expression is absolutely required for pituitary organogenesis [28]. Expression of *Lhx3* is not significantly altered in *Rx−/−* embryos (Fig. 2B, D), when compared to wild type embryos (Fig. 2A, C), in spite of the fact that the anterior pituitary is severely dysmorphic in *Rx−/−* embryos. This indicates that the program of anterior pituitary organogenesis has been initiated.

*Fgf10* is a diagnostic marker of posterior pituitary development [23]. In wild type embryos, it is expressed in the ventral hypothalamus and the infundibulum (Fig. 2E, G). In *Rx −/−* embryos, *Fgf10* expression is initially quite normal at E9.5 (Fig. 2F), but later, at E10.5 is severely downregulated (Fig. 2H). No cells can be found that express this gene at high levels typical of posterior pituitary development.

The homeobox gene *Optx2 (Six6)* is co-expressed with *Rx e*arly in development in both the ventral diencephalon and the optic vesicle In addition this gene is expressed in the head surface ectoderm [29]–[32]. In *Rx*-deficient embryos, *Optx2* expression is completely lost in the optic vesicle/cup at E9.5 (Fig. 2I,J). Strong reduction of *Optx2* expression in the head of *Rx−/−* embryos is already evident at E8.5 (Fig. 2K,L). In both stages in Rx−/− embryos *Optx2* is expressed only in the surface ectoderm, but not in the neuroectoderm. At E10.5 (Fig. 2M,N) Optx2 is not expressed in the optic vesicle or in the ventral hypothalamus. These experiments, taken together with the observation that *Rx* is expressed in *Optx2* mutant mice [33], indicate that *Optx2* is genetically downstream of *Rx* and is potentially a direct regulatory target for *Rx*. However, a direct regulation of *Optx2* expression by *Rx* remains to be demonstrated.

**Figure 3:**
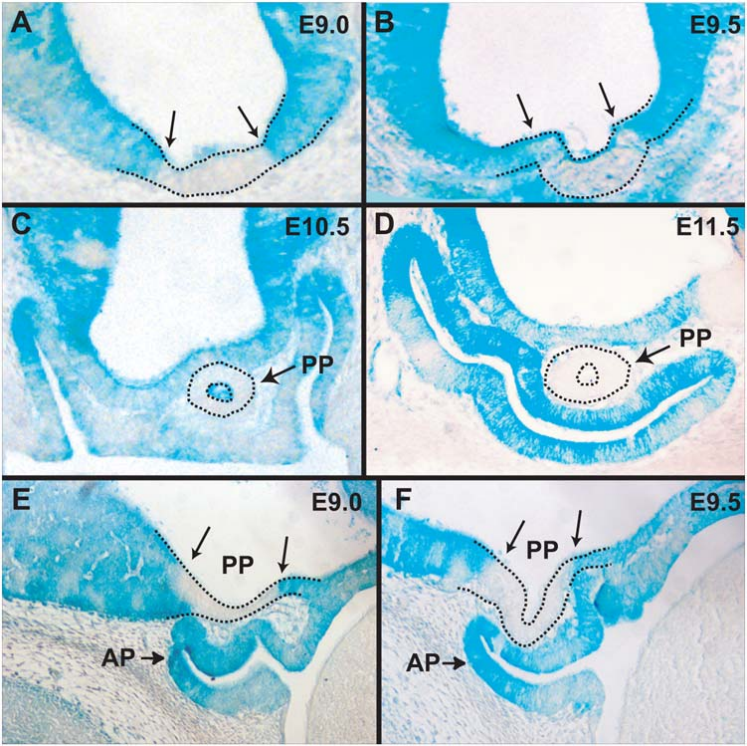
Formation of the posterior pituitary in chimeric embryos. (A) Section through an E9.0 chimeric embryo demonstrating a region free of *Rx*-deficient cells in the ventral hypothalamus. Two arrows indicate the sharp boundaries between the wild type cells (white) and *Rx−/−* cells (blue). (B) Section through an E9.5 chimeric embryo demonstrating that the evaginating posterior pituitary does not contain any *Rx*-deficient cells. Two arrows indicate the sharp boundaries between the wild type cells and *Rx−/−* cells. (C) Section through an E10.5 chimeric embryo showing that the tubular posterior pituitary is devoid of *Rx−/−* cells. (D) Section through an E11.5 chimeric embryo demonstrating that the posterior pituitary is devoid of *Rx−/−* cells. (E) Sagittal section of an E9.0 chimeric embryo visualizing the *Rx−/−* cell free region in the ventral hypothalamus. (F) Sagittal section of an E9.5 chimeric embryo showing that the evaginating posterior pituitary does not contain *Rx−/−* cells. AP - anterior pituitary, PP – posterior pituitary.

Due to the lack of a visible posterior pituitary and the changes in gene expression in the ventral hypothalamus of *Rx−/−* embryos, we analyzed the ability of *Rx−/−* cells to contribute to the formation of the posterior pituitary in chimeric embryos. We found that *Rx−/−^lacZ/+^* cells cannot contribute to the formation of the posterior pituitary. As early as E9.0, there is a distinct region with sharp boundaries in the ventral hypothalamus that is devoid of blue, *Rx*-deficient cells (Fig. 3A,E). This region undergoes evagination (Fig. 3B,F) and later forms the posterior pituitary, consisting exclusively of wild type cells (Fig. 3C,D). This observation suggests that *Rx* function is required cell-autonomously for the formation of the field of posterior pituitary progenitor cells even before the morphogenesis of the posterior pituitary begins. The similarities between the cell autonomous requirements for *Rx* function between the presumptive neuroectoderm of the retina and posterior pituitary, as well as the lack of *Optx2* expression in both tissues in *Rx*-deficient embryos, gives support to the hypothesis that the development of the retina and the posterior pituitary is regulated by similar developmental and molecular mechanisms.

## Discussion

### Cell-autonomous requirement for *Rx* function in the retina and posterior pituitary

We have presented evidence that in mice *Rx* activity is required cell autonomously for the formation of the neuroretina, retina pigment epithelium and the distal optic stalk. *Rx*-deficient cells cannot be found in these structures in chimeric embryos. At the beginning of evagination, the presumptive ocular neuroectoderm of chimeric embryos contains almost exclusively wild type cells, indicating that *Rx−/−* cells are eliminated from the presumptive retinal neuroectoderm before the onset of retinal morphogenesis. This suggests that *Rx−/−* cells never behave like, or are recognized, as retinal progenitor cells. Segregation of wild type and *Rx−/−* cells can be observed in the proximal optic stalk. In this transitional zone between the optic stalk and the brain, wild type and *Rx*-deficient cells can be observed next to each other forming columns of cells segregated by their genetic makeup. The wild type and *Rx−/−* columns of cells have different morphology, presumably because of different morphogenetic behavior. The wild type columns are thinner presumably because these cells are undergoing convergent extension typical of optic stalk formation. The *Rx−/−* cells do not appear to undergo convergent extension [2], [11], [14].

Retinas in chimeric embryos show a remarkable degree of autonomy. Even in embryos where most of the surrounding cells are mutant, retinal development appears to be normal, i.e. the optic cup forms a normal RPE, neuroretina and optic stalk. Only in cases when very few wild type cells are available to form a retina, the differentiation of the optic cup is abnormal. In this case, the entire retina and optic stalk differentiate into RPE. This is interesting, as it indicates that the abnormal differentiation of retinal cells is not due to the genetic makeup of the retinal cells, but rather due to the small size of the retina. A likely explanation for this phenomenon is provided by the observations of Cho and Cepko [34]. They found that during chick eye development, a yet unknown mechanism induces expression of *Wnt2b* in the surface ectoderm dorsally to the evaginating optic cup. Somewhat later, *Wnt2b* expression spreads to the adjacent dorsal optic cup, which then becomes RPE. It is possible that when the evaginating optic cup is very small, the *Wnt2b*-inducing signal transverses the entire optic cup and converts it to RPE. This retina, consisting entirely of RPE cells, cannot induce lens formation suggesting that neuroretina or neuroretina-specific gene expression is necessary for lens induction. Alternatively, the evaginating retinal neuroectoderm is too small to ever contact the surface ectoderm and therefore the inductive signals from the optic vesicle never reach the surface ectoderm.

The posterior pituitary gland of chimeric embryos does not contain *Rx*-deficient cells showing that *Rx* function is also required cell-autonomously for the formation of the posterior pituitary. Similar to the ocular neuroectoderm, the neuroectoderm of the presumptive posterior pituitary is depleted of *Rx−/−* cells prior to the evagination of the posterior pituitary. This shows that *Rx* function is required for the establishment of a field of posterior pituitary progenitor cells, indicating that the development of the retina and posterior pituitary is regulated by a similar developmental mechanism.

Our findings in mouse embryos agree with the observation in medaka and zebrafish that cells mutant for *Rx* function cannot participate in the morphogenesis of the optic vesicle [13], [25]–[27], [35]. Furthermore, our observation taken together with the findings of Stigloher and coworkers [26] that zebrafish *Rx3* controls segregation of cells in the zebrafish forebrain, provide evidence that *Rx* genes control the formation of fields of cells with specific properties and distinct boundaries. This is an important observation, as it provides a possible explanation for the mechanism involved in the initial stages of evagination of the optic vesicle and the posterior pituitary. It is likely that during development, the different fields of cells react differently to the pressure generated by the growth of the neuroectoderm, leading to its buckling [36]. As a result, specific groups of progenitor cells evaginate from the neuroectoderm.

In summary, there appear to be two different reasons why *Rx−/−* cells cannot contribute to the formation of the retina and posterior pituitary in *Rx−/−* embryos and chimeras. It appears that the progenitor of the retina and the posterior pituitary are not specified in *Rx−/−* animals, and consequently the retina and the posterior pituitary do not form. However, it is difficult to exclude the possibility a very few cells become progenitor cells. Nevertheless, even if some cells are specified, *Rx* function is required for the expression of *Optx2* [this paper], [ reference 37]–[39], a gene that is required for their proliferation [40], [41]. A disproportionably small number of cells might not be able to form an ocular neuroectoderm or survive. In chimeric embryos, *Rx−/−* cells are not recognized as progenitor cells of the retina and the posterior pituitary and are eliminated from these structures.

**Figure 4:**
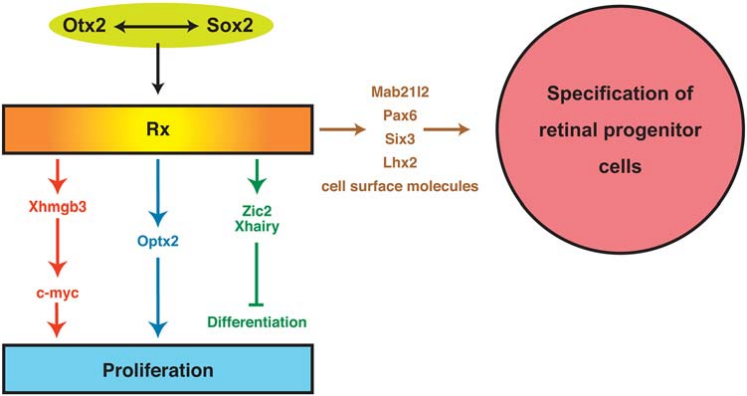
Schematic diagram of regulatory interactions taking place during retinal development. This simplified view based on observations of several investigators shows that *Otx2* and *Sox2* activate expression of *Rx*. *Rx* then directly or indirectly activates a battery of genes that are involved in the proliferation as well as the morphogenesis and early development of the retinal neuroectoderm. At least three independent pathways are involved in the activation of proliferation in *Rx* expressing cells. The first pathway is mediated by *Xhmgb3*, the second utilizes the function of *Optx2* and the third takes advantage of the anti-differentiation activity of *Zic2/Xhairy*. At the same time, *Rx* expressing cells also activate several transcriptional factors like *Pax6*, *Mab21/2*, *Six3* and *Lhx2* that are necessary for the correct development of the optic vesicle. Finally, our data suggests that *Rx* activates genes encoding specific cell surface molecules necessary for the segregation of *Rx*-expressing cells.

The current understanding of regulatory interactions in which *Rx* plays a key role with the focus on retinal development is depicted in figure 4. It is mostly based on experiments in *Xenopus*, as to date a more detailed analysis was performed in this species than in mouse. As demonstrated by Danno and coworkers [42], *Rx* is activated in the forebrain by the coordinate action of *Otx2* and *Sox2*. *Rx* in turn activates or upregulates expression of several proteins that are essential for the specification and early development of the retinal neuroectoderm. Genes included in this category are transcription factors like *Pax6*, *Six3*, *Lhx2* and *Mab21l2*. At the same time *Rx* stimulates the proliferation of these cells through several apparently independent pathways. The first pathway begins with the activation of the high mobility group B3 gene *Xhmgb3* followed by the activation of *c-myc* [39]. The second pathway begins with the activation of *Optx2* transcription [this paper], [ reference 37]–[39], a gene that is known to be an activator of proliferation [40], [41]. In addition, *Rx* also activates *Zic2* and *Hairy*, two genes that inhibit the differentiation of neural cells and keep them in proliferative state. Finally, the segregation of cells in chimera based on the genetic make up suggests that genes encoding specific cell surface molecules are also activated at this time. However, the identity of these molecules remains to be elucidated.

## Materials and Methods

### *Rx−/−;Gt(ROSA)26Sor/J* ES cells

To generate *Rx−/−;Gt(ROSA)26Sor/J* ES cells, the *Rx+/−* mice were first crossed into the *Gt(ROSA)26Sor/J* mouse line [43] that ubiquitously expresses ß-galactosidase. Blastocysts from a cross *Rx+/−;Gt(ROSA)26Sor/J x Rx+/−;Gt(ROSA)26Sor/J* were used to make ES cells. ES cells identified as *Rx+/+;Gt(ROSA)26Sor/J* and *Rx−/−;Gt(ROSA)26Sor/J* were used for further experiments.

### Chimeras

To generate chimeric embryos, blastocysts from C57BL/6-J females were harvested at 3.5 d.p.c. and were injected with *Rx−/−;Gt(ROSA)26Sor/J* ES cells [44]. These embryos were then transferred into the uterine horns of pseudopregnant females [45]–[47]. Embryos were analyzed at different developmental stages for the presence of *Rx−/−* cells in the eye. The *Rx−/−;Gt(ROSA)26Sor/J* cells were visualized by LacZ staining and appeared blue. The wild type cells were white. In control embryos, wild type cells were white and the *Gt(ROSA)26Sor/J* cells that were wild type for *Rx*, were blue. Chimeric embryos were harvested at different stages of embryonic development and the ES cell contribution of *Rx+/+^lacZ/+^* or *Rx−/−^lacZ/+^* cells was determined by the proportion of blue cells in the whole embryo. In our experiments, we evaluated chimeras with moderate or high contribution of *Rx−/−^lacZ/+^* cells.

## References

[1] Casarosa S, Andreazzoli M, Simeone A, Barsacchi G (1997). Xrx1, a novel Xenopus homeobox gene expressed during eye and pineal gland development.. Mech Dev.

[2] Mathers PH, Grinberg A, Mahon KA, Jamrich M (1997). The Rx homeobox gene is essential for vertebrate eye development.. Nature.

[3] Furukawa T, Kozak CA, Cepko CL (1997). rax, a novel paired-type homeobox gene, shows expression in the anterior neural fold and developing retina.. Proc Natl Acad Sci U S A.

[4] Bailey TJ (2004). Regulation of vertebrate eye development by Rx genes.. Int J Dev Biol.

[5] Loosli F (2003). Loss of eyes in zebrafish caused by mutation of chokh/rx3.. EMBO Rep.

[6] Loosli F (2001). Medaka eyeless is the key factor linking retinal determination and eye growth.. Development.

[7] Ohuchi H, Tomonari S, Itoh H, Mikawa T, Noji S (1999). Identification of chick rax/rx genes with overlapping patterns of expression during early eye and brain development.. Mech Dev.

[8] Voronina VA (2004). Mutations in the human RAX homeobox gene in a patient with anophthalmia and sclerocornea.. Hum Mol Genet.

[9] Tucker P (2001). The eyeless mouse mutation (ey1) removes an alternative start codon from the Rx/rax homeobox gene.. Genesis.

[10] Swindell EC (2008). Eye formation in the absence of retina.. Dev Biol.

[11] Swindell EC (2006). Rx-Cre, a tool for inactivation of gene expression in the developing retina.. Genesis.

[12] Brownell I, Dirksen M, Jamrich M (2000). Forkhead Foxe3 maps to the dysgenetic lens locus and is critical in lens development and differentiation.. Genesis.

[13] Winkler S, Loosli F, Henrich T, Wakamatsu Y, Wittbrodt J (2000). The conditional medaka mutation eyeless uncouples patterning and morphogenesis of the eye.. Development.

[14] Zhang L, Mathers PH, Jamrich M (2000). Function of Rx, but not Pax6, is essential for the formation of retinal progenitor cells in mice.. Genesis.

[15] Sheng HZ (1997). Multistep control of pituitary organogenesis.. Science.

[16] Rosenfeld MG (2000). Multistep signaling and transcriptional requirements for pituitary organogenesis in vivo.. Recent Prog Horm Res.

[17] Hermesz E, Mackem S, Mahon KA (1996). Rpx: a novel anterior-restricted homeobox gene progressively activated in the prechordal plate, anterior neural plate and Rathke's pouch of the mouse embryo.. Development.

[18] Schwind J (1928). The development of the hypophysis cerebri of the albino rat.. Amer J Anat.

[19] Scully KM, Rosenfeld MG (2002). Pituitary development: regulatory codes in mammalian organogenesis.. Science.

[20] Ericson J, Norlin S, Jessell TM, Edlund T (1998). Integrated FGF and BMP signaling controls the progression of progenitor cell differentiation and the emergence of pattern in the embryonic anterior pituitary.. Development.

[21] Takuma N (1998). Formation of Rathke's pouch requires dual induction from the diencephalon.. Development.

[22] Treier M (1998). Multistep signaling requirements for pituitary organogenesis in vivo.. Genes Dev.

[23] Ohuchi H (2000). FGF10 acts as a major ligand for FGF receptor 2 IIIb in mouse multi-organ development.. Biochem Biophys Res Commun.

[24] Kondoh H (2000). Zebrafish mutations in Gli-mediated hedgehog signaling lead to lens transdifferentiation from the adenohypophysis anlage.. Mechanisms of Development.

[25] Rembold M, Loosli F, Adams RJ, Wittbrodt J (2006). Individual cell migration serves as the driving force for optic vesicle evagination.. Science.

[26] Stigloher C (2006). Segregation of telencephalic and eye-field identities inside the zebrafish forebrain territory is controlled by Rx3.. Development.

[27] Rojas-Munoz A, Dahm R, Nusslein-Volhard C (2005). chokh/rx3 specifies the retinal pigment epithelium fate independently of eye morphogenesis.. Dev Biol.

[28] Sheng HZ (1996). Specification of pituitary cell lineages by the LIM homeobox gene Lhx3.. Science.

[29] Jean D, Bernier G, Gruss P (1999). Six6 (Optx2) is a novel murine Six3-related homeobox gene that demarcates the presumptive pituitary/hypothalamic axis and the ventral optic stalk.. Mech Dev.

[30] Toy J, Sundin OH (1999). Expression of the optx2 homeobox gene during mouse development.. Mech Dev.

[31] Toy J, Yang JM, Leppert GS, Sundin OH (1998). The optx2 homeobox gene is expressed in early precursors of the eye and activates retina-specific genes.. Proc Natl Acad Sci U S A.

[32] Lopez-Rios J, Gallardo ME, Rodriguez de Cordoba S, Bovolenta P (1999). Six9 (Optx2), a new member of the six gene family of transcription factors, is expressed at early stages of vertebrate ocular and pituitary development.. Mech Dev.

[33] Li X, Perissi V, Liu F, Rose DW, Rosenfeld MG (2002). Tissue-specific regulation of retinal and pituitary precursor cell proliferation.. Science.

[34] Cho SH, Cepko CL (2006). Wnt2b/beta-catenin-mediated canonical Wnt signaling determines the peripheral fates of the chick eye.. Development.

[35] Kennedy BN (2004). Zebrafish rx3 and mab21l2 are required during eye morphogenesis.. Dev Biol.

[36] Volokh KY (2006). Tissue morphogenesis: a surface buckling mechanism.. Int J Dev Biol.

[37] Zuber ME, Gestri G, Viczian AS, Barsacchi G, Harris WA (2003). Specification of the vertebrate eye by a network of eye field transcription factors.. Development.

[38] Andreazzoli M (2003). Xrx1 controls proliferation and neurogenesis in Xenopus anterior neural plate.. Development.

[39] Terada K, Kitayama A, Kanamoto T, Ueno N, Furukawa T (2006). Nucleosome regulator Xhmgb3 is required for cell proliferation of the eye and brain as a downstream target of Xenopus rax/Rx1.. Dev Biol.

[40] Bernier G (2000). Expanded retina territory by midbrain transformation upon overexpression of Six6 (Optx2) in Xenopus embryos.. Mech Dev.

[41] Zuber ME, Perron M, Philpott A, Bang A, Harris WA (1999). Giant eyes in Xenopus laevis by overexpression of XOptx2.. Cell.

[42] Danno H (2008). Molecular links among the causative genes for ocular malformation: Otx2 and Sox2 coregulate Rax expression.. Proc Natl Acad Sci U S A.

[43] Friedrich G, Soriano P (1991). Promoter traps in embryonic stem cells: a genetic screen to identify and mutate developmental genes in mice.. Genes Dev.

[44] Nagy A (2000). Manipulating the Mouse Embryo.

[45] Gardner RL, Davies TJ (2000). Mouse chimeras and the analysis of development.. Methods Mol Biol.

[46] Gardner RL (1975). Analysis of determination and differentiation in the early mammalian embryo using intra- and interspecific chimeras.. Symp Soc Dev Biol.

[47] Gardner RL (1968). Mouse chimeras obtained by the injection of cells into the blastocyst.. Nature.

